# Structural and Functional Properties of Porous Corn Starch Obtained by Treating Raw Starch with AmyM

**DOI:** 10.3390/foods12173157

**Published:** 2023-08-22

**Authors:** Lei Zhang, Lingli Zhong, Peiwen Wang, Lei Zhan, Yunzhen Yangzong, Tianqi He, Yi Liu, Dongmei Mao, Xianfeng Ye, Zhongli Cui, Yan Huang, Zhoukun Li

**Affiliations:** Key Laboratory of Agricultural Environmental Microbiology, Ministry of Agriculture and Rural Affairs, College of Life Sciences, Nanjing Agricultural University, Nanjing 210095, China; 2017116060@njau.edu.cn (L.Z.); 2022216012@stu.njau.edu.cn (L.Z.); 2022116072@stu.njau.edu.cn (P.W.); 10121101@stu.njau.edu.cn (L.Z.); 10121109@stu.njau.edu.cn (Y.Y.); 10121212@stu.njau.edu.cn (T.H.); 10121106@stu.njau.edu.cn (Y.L.); maodongmei@njau.edu.cn (D.M.); czl@njau.edu.cn (Z.C.); huangyan@njau.edu.cn (Y.H.)

**Keywords:** porous starch, raw starch digesting amylase, chain rearrangement, starch digestion

## Abstract

Porous starch is attracting considerable attention for its high surface area and shielding ability, properties which are useful in many food applications. In this study, native corn starch with 15, 25, and 45% degrees of hydrolysis (DH-15, DH-25, and DH-45) were prepared using a special raw starch-digesting amylase, AmyM, and their structural and functional properties were evaluated. DH-15, DH-25, and DH-45 exhibited porous surface morphologies, diverse pore size distributions and pore areas, and their adsorptive capacities were significantly enhanced by improved molecular interactions. Structural measures showed that the relative crystallinity decreased as the DH increased, while the depolymerization of starch double helix chains promoted interactions involving disordered chains, followed by chain rearrangement and the formation of sub-microcrystalline structures. In addition, DH-15, DH-25, and DH-45 displayed lower hydrolysis rates, and DH-45 showed a decreased *C*_∞_ value of 18.9% with higher resistant starch (RS) content and lower glucose release. Our results indicate that AmyM-mediated hydrolysis is an efficient pathway for the preparation of porous starches with different functionalities which can be used for a range of applications.

## 1. Introduction

Starch is a complex branched-chain homopolymer of glucose that is widely used in the food, feed, fuel, chemical, materials, pharmaceutical, and environments industries due to its nontoxicity, utilizability, and biodegradability [1]. The water-insoluble semi-crystalline starch granules in plants are mainly composed of amylopectin and amylose, which directly determine the properties of the starch and its potential applications. Physical, chemical, and enzymatic hydrolysis techniques have been used to modify starch structures in order to improve their potential properties, including digestion, gelatinization, viscosity, and solubility, and thereby expand their possible uses [2]. Unlike heating or gelatinization under high temperatures, the modification of raw starch below the gelatinization temperature represents an alternative energy-conserving means of improving the properties of starch materials [3].

Among granule-based starch products, porous starch with abundant micro-sized pores has attracted considerable attention due to its high specific surface area and potential molecular interactions, which make it suitable for a wide variety of applications, e.g., as an absorbent, carrier, protector [4], or multifunctional biomaterial [5]. Improvements in noodle cooking and noodle texture quality have also been achieved via the incorporation of porous starch [6]. The uniqueness and the advantages of porous starch have driven additional studies with a focus on their preparation and related physicochemical properties. Among the various physical, chemical, and biological preparation methods, enzymatic hydrolysis has received considerable attention because pores of different sizes can be produced under mild conditions without changing the shape of the starch granules. Currently, α-amylase, β-amylase, glucoamylase, glycosyltransferase, and isoamylase have been shown to exhibit activity toward starch granules (Table S1), and extensive research involving α-amylase and glucoamylase in both single and combined applications in porous starch preparations has been carried out [2,7]. However, different types of enzymes and different hydrolysis conditions lead to different pore-size distributions, internal order structures, and physicochemical properties. Porous maize starch prepared using α-amylase and glucoamylase treatments exhibited a 50% increase in oil absorption, and the particle distribution width increased from 1.1 to 1.56 μm [5]. Sequential α-amylase and glucoamylase produced abundant mesopores (2–50 nm), and the oil absorbency increased by 9.2 times, whereas a sequential glycosyltransferase and branching enzyme treatment produced many more macropores (>50 nm), and the oil absorbency increased by 12.8 times [8]. The hydrolysis of corn starch using barley malt amylases produced visible outer pinholes and disrupted the interior crystallized region [9], while a sequential α-amylase and branching enzyme treatment increased the relative crystallinity by 4%, with minor effects on the in vitro digestion of raw starch [10]. These results provide direct evidence of the different functional properties of porous starch that can be produced using various enzyme treatments. Hence, the utilization of special raw starch-digesting enzymes (RSDEs) in the preparation of porous starch is of significance for the efficient utilization of raw starch.

Native starch exists naturally as insoluble semi-crystalline granules of different particle sizes and shapes which are assembled by amylose and amylopectin. The insoluble surface and hierarchical interior structures are able to prevent access-binding and the action of amylase [11]. Hence, although enzymatic modification has become the primary method for preparing porous starches, amylolytic enzymes which have the ability to efficiently digest raw starch without pretreatment are still limited by the compactness and crystallinity of the granules, and a single application of α-amylase has been shown to provide a lower ability to hydrolyze tuber starches with slight fissures, shallow pores, and a 23% degree of hydrolysis [12]. In addition, a previous study has shown that the pasting property of maltogenic α-amylase-treated starch is clearly linearly correlated with the degree of hydrolysis [13], and additional correlative studies exist linking the fine structural parameters of starch to texture or digestibility [14], indicating a relationship between the molecular structures within raw starch granules and their functions. However, the underlying properties need to be further explored.

In a previous study, we identified a novel maltohexaose-forming α-amylase—AmyM—with high starch liquefaction and maltooligosaccharide (MOS) formation ability [15]. A further study found that AmyM is also an efficient RSDE with robust substrate and product tolerance, and a signal enzyme treatment resulted in the formation of surface pores and the production of MOSs and dextrin with a high degree of hydrolysis (95%) [3]. An application evaluation showed that AmyM has positive effects on bread quality and digestion (i.e., increased slowly digestible starch content) [16,17], indicating that a distinct structural changes occurred in the starch chains during particle decomposition. In this study, the effects of AmyM modification on the structure and functional properties of raw corn starch were investigated. This study will contribute to the development of a novel approach to the preparation of porous starch. 

## 2. Materials and Methods

### 2.1. Materials

The corn starch was purchased from Yuanye Biotechnology Co., Ltd. (Shanghai, China). The maltohexaose-forming α-amylase (AmyM), derived from *Corallococcus* sp. EGB, was prepared in *Pichia pastoris* GS115 using a feed-batch strategy involving waste molasses and industrial glucose [18]. Commercial pancreatin and amyloglucosidase were purchased from Sigma Chemical (St. Louis, MO, USA). All chemicals and other reagents used in this study were of analytical grade and were purchased from Sinopharm Chemical Reagent Co., Ltd. (Shanghai, China).

### 2.2. Modification of Native Corn Starch Using AmyM and SEM Observation

#### 2.2.1. AmyM Hydrolysis of Corn Starch Granules

The corn starch (10% *v*/*w*) was dispersed in water and then treated with the prepared AmyM (0.5 U/mg dosage) at 45 °C and 180 rpm with different reaction times. During the hydrolytic process, the reducing sugars from the reaction system were identified using the 3′5-dinitrosalicylic acid (DNS) method [19], and the degree of hydrolysis (% DH) was calculated as the ratio of the amount of soluble sugars from the starch hydrolysis to the initial mass of starch. Subsequently, the reaction was stopped by increasing the pH of reaction system to 11.0 using sodium hydroxide, and then by reducing the pH back to 7.0 using HCl. The hydrolysate was centrifuged at 6000 rpm for 15 min, and the starch residues were washed with deionized water six times. The sediments were freeze dried to obtain modified starch samples with different DH values (DH-15, DH-25, and DH-45).

#### 2.2.2. SEM Observations

To observe the morphological features, the dried starch samples with DH values of 15, 25, and 45 were fixed in glutaraldehyde and a scanning electron microscope (SEM) analysis was performed using an SU8010 microscope (Hitachi, Japan); representative images were obtained. Untreated native corn starch was used as a control. The samples were coated with gold prior to observation and then examined at an accelerating voltage of 5 kV and 2500× magnification [20].

### 2.3. Characteristics of the Modified Starch with Porous Morphology

#### 2.3.1. Pore Diameter

To determine the pore diameter of modified corn starch, the starch samples were investigated using the Brunauer–Emmett–Teller method (BET, Autosor-iQ, Quantanchrome INSTRUMENTS, Boynton, FL, USA) with liquid nitrogen adsorption at −196 °C and relative pressure ranging from 0.01 to 1.0. The adsorbent was porous starch, and the adsorbent mass was nitrogen. The BJH equation was used to determine the surface area, volume, and average pore diameter. 

#### 2.3.2. Water Solubility and Swelling Power

To determine the water solubility and swelling power, starch suspensions (2% concentration) were heated in a water bath shaker at 85 °C for 30 min, and, after cooling to room temperature, centrifuged at 3000 rpm for 15 min. The precipitate was weighed, and the supernatant was dried at 105 °C until it reached a constant weight. The water solubility index (WSI) and swelling power index were calculated according to the method described in [21]. 

#### 2.3.3. Gel Consistency and Transparency

The gel consistency was determined according to the method outlined in [22] (with minor modifications). Each porous starch sample (100 mg) was weighed in a 25 mL test tube, to which 0.2 mL thymol blue solution (0.025% thymol blue in 85% ethanol) was added, and the contents were then mixed for 10 s. To terminate the reaction, 2 mL KOH (0.2 M) was added and the tube was covered with a marble and boiled for 7 min. During the boiling treatment, the starch slurry level was maintained at one third of the tube’s length. The tube was then cooled at room temperature for 5 min and subjected to ice water treatment for 20 min, after which the tube was placed horizontally on a table for 1 h. The gel consistency was measured as the extent to which the gel spread. In addition, each starch sample (1% *w*/*v*) was prepared at 80 °C for 30 min under constant stirring, and the transparency of each starch sample was determined by measuring its light transmittance at OD_650_.

### 2.4. Structural Properties of the Porous Starch

#### 2.4.1. Crystallinity

The crystallinity of the prepared DH-15, DH-25, and DH-45 samples was analyzed using a D2 PHASER X-ray diffractometer (Bruker, GER) operating at 40 kV and 40 mA with Cu Kα radiation (λ = 0.154 nm). The samples were equilibrated over a saturated NaCl solution until the water content reached about 9%, at which point they were packed tightly in a glass cell and scanned from 5–35° (2θ) at a rate of 2°/min and a step size of 0.02°. The relative reflection areas in the crystalline and amorphous portions were distinguished via the straight line joining the points of intensity at the 35° and 5° diffraction angles, and the relative crystallinity (RC, %) of the starch samples, i.e., the ratio of the crystalline area to the total area between 5° and 35° (2θ) [23], was calculated using Jade 6.0 software (Materials Dta Inc., Livermore, CA, USA).

#### 2.4.2. Fourier Transform Infrared Spectroscopy (FTIR)

The FTIR spectra of the dried starch samples was obtained using a Thermo Nicolet Nexus FTIR spectrometer (Thermo Scientifc, Waltham, MA, USA) equipped with a single-reflection diamond attenuated total reflection (ATR) crystal and a mercury–cadmium–telluride (MCT) detector. The spectra were scanned within a range of 500–4000 cm^−1^ and with a resolution of 4 cm^−1^. The collected spectra were automatically baseline-corrected using OMNIC 8.0 (Thermo Nicolet, Waltham, MA, USA), and the region ranging from 1200 to 900 cm^−1^ was deconvoluted with a half bandwidth of 20 cm^−1^ and an enhancement factor of 1.8. Bands at 1022, 1047, and 995 cm^−1^ were detected in the spectra, and the ratios of the peak intensity of absorption bands at 1047/1022 cm^−1^ and 1022/995 cm^−1^ were used to characterize the short-range molecular order of starch samples, as well as the number of double helices [24].

### 2.5. Functional Properties of the Porous Starch

#### 2.5.1. Thermal Properties

To analyze the thermal properties of the porous starch, 3 mg samples were weighted in stainless steel pans and mixed with 9 μL water with a starch/water ratio of 1:3 (*w*/*v*). The sealed pans were kept at room temperature overnight for equilibration, following which they were scanned from 20 to 100 °C at a heating rate of 10 °C/min using a differential scanning calorimeter (DSC7000, Seiko Instrument, Inc., Chiba, Japan) equipped with a thermal analysis data station. The onset temperature (*T*_o_), peak temperature (*T*_p_), conclusion temperature (*T*_c_), and enthalpy (Δ*H*) were calculated from the DSC curves obtained using the data-recording software [25]. 

#### 2.5.2. Digestion Evaluation and Expected Glycemic Index of the Porous Starch

The in vitro digestion of the raw starch and the DH-15, DH-25, and DH-45 samples was analyzed using the method described by Gularte and Rosell [26] (with minor modifications). Briefly, 100 mg starch (dry weight) was dissolved in 4 mL of 0.1 M sodium maleate buffer (pH 6.9, 1 mM CaCl_2_) with pancreatin (Type VI-B, ≥10 U/mg solid, Sigma Chemical, St. Louis, MO, USA) (final activity: 6 U/mL) in a shaking water bath at 37 °C. During the enzymatic reaction, starch sample aliquots (0.2 mL) were taken at 10, 20, 30, 40, 60, 90, 120, 150, and 180 min, and these were mixed with 0.2 mL ethanol (96% *v*/*v*) to stop the reaction. The mixture was centrifuged at 10,000× *g* for 5 min, and the precipitate was washed three times with 50% ethanol (200 mL). All the produced supernatants were pooled together for the subsequent amyloglucosidase treatment. Mixtures containing 0.1 mL supernatant, 0.85 mL buffer (0.1 M sodium acetate, pH 5.2, 1 mM CaCl_2_), and 50 μL amyloglucosidase (10 U/mL) were incubated at 40 °C for 30 min in a shaking water bath. The amount of released glucose was measured using a D-glucose assay kit (GOPOD format, Megazyme), and the final starch hydrolysis curve was plotted according to the starch hydrolysis (%) against time. The formula used to calculate the digestibility of the starch in vitro as well as the method used to produce the digestograms of starch hydrolysis are outlined in [26], and the maximum hydrolysis extent (*C*_∞_), kinetic constant (*k*), area under the hydrolysis curve after 180 min (AUC 180), hydrolysis index (HI), and expected glycemic index (eGI) were calculated. Additionally, the resistant starch (RS) content was quantified according to the established method [26,27].

### 2.6. Statistical Analysis

All data were subjected to one-way ANOVA tests using SPSS Version 10.0. ANOVA mean comparisons were performed according to the least significant difference (LSD). Differences with *p*-values of <0.05 were considered statistically significant. All experiments in the study were performed in triplicate and produced similar results.

## 3. Results and Discussion

### 3.1. Modification of Raw Corn Starch with Porous Morphology Using AmyM

**Figure 1:**
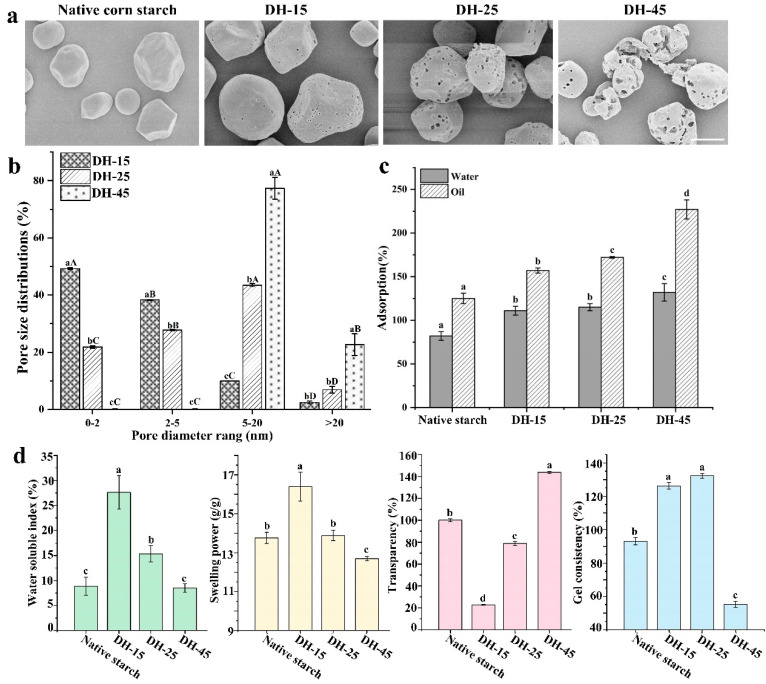
Observed morphologies of the porous corn starch samples and analysis of their related characteristics. (**a**) Scanning electron micrograph of native corn starch, DH–15, DH–25, and DH–45 prepared via AmyM modification with different degrees of hydrolysis. Scale bar: 10 μm. (**b**) Particle size distributions of the starch samples. The average particle size ranged from 2 to 20 nm, and particles were measured in triplicate. Uppercase letters represent the treatments with the same DH values but different sizes, and lowercase letters represent the treatments with the same sizes but different DH values. (**c**) Adsorption capacity of the porous starches with water and oil as additives. (**d**) Characteristics of the porous corn starches (water solubility, swelling power, transparency, and gel consistency). Error bars denote st. dev. (SD), and values with different letters indicate statistically significant differences (*p* < 0.05).

The enzymolysis of the raw corn starch was performed at 45 °C to obtain AmyM-modified starch samples with different degrees of hydrolysis (DH-15, DH-25, and DH-45), and SEM observations were performed to obtain visualizations of their surface morphologies. As is shown in Figure 1a, after the action of the AmyM, the surface of the corn starch granules formed a uniform porous structure. The surface apertures of the DH-15 starch were small and shallow compared with the large and visible pore sizes of the DH-25 starch. In addition, DH-45 showed deep aperture structures with slight particle decomposition. In our previous study, DH-20 and DH-50 starch samples were prepared via the hydrolysis of raw corn starch using AmyM, and we observed that DH-50 exhibited a decomposed morphology without intact granules, while DH-20 showed inhomogeneous holes [3]. Both of these starch samples therefore differed from the DH-25 and DH-45 samples used in this study (Figure 1a). This is why we adjusted the DH (15, 25, and 45) of the native corn starch using AmyM and visualized the diversity of the surface morphologies. The pore size distributions of the starch samples were also analyzed. The DH-15 and DH-25 samples had average pore diameters of 0–2 nm and 2–5 nm, respectively, while the DH-45 sample showed increased pore sizes (average diameter: 5–20 nm; some pores > 20 nm) (Figure 1b). Pores less than 2 nm in diameter are classified as micropores, and pores that are 2–50 nm in dimeter are classified as mesopores [2]. Native corn starch granules with smaller pores have been observed in other RSDEs-treated samples [28]. The surface hydrophobicity and fine molecular organization of raw starch granules directly restricts enzymatic hydrolysis at the solid–liquid interface [29]. Hence, grinding pretreatment or magnetic stirring is used to increase the granules’ water-binding capacity [30], higher temperatures (≥60 °C) are used to altering starch–amylase interactions [31], and synergistic hydrolysis methods with combined amylases [32] have been developed to prepare porous starch granules. The hydrolysis of native corn starch using AmyM without any destructive treatment represents an alternative way to prepare porous starch under low temperatures and at low dosages. The efficient conversion of native starch granules into soluble content with a 95% hydrolysis rate [3] and a porous shape within the gluten networks of native dough [17] indicates that AmyM harbors distinct hydrolytic characteristics that facilitate the preparation of porous starches with different properties. Abundant and randomly distributed nanoscale holes were observed in the lamellar structures of DH-45 (Figure 1a). These results indicate that AmyM-mediated starch granule hydrolysis is an efficient pathway for the preparation of porous starch with various potential functionalities.

To further clarify the porosity characteristics of the AmyM-modified raw starch samples, their adsorption, swelling power, solubility, and gel consistency were analyzed. Two different liquids (water and oil) were used, and the adsorption properties of DH-15, DH-25, and DH-45 were significantly improved after the enzyme treatment (Figure 1c). DH-15 and DH-25 showed increased water and oil absorption (Figure 1c). DH-45, which exhibited slight particle decomposition, had a higher adsorption capacity than DH-25 and large visible pores (Figure 1c). The porous starch prepared using commercial α-amylase had higher oil absorption but the lower water absorption, and this may contribute to both the deep and large pore channels and the excessive decomposition which weakened the water and oil adsorption capacity of the porous starches [5]. Sequential α-amylase and glucoamylase hydrolysis produced abundant mesopores on the starch granules (2–50 nm), and the oil absorption accordingly increased by 9.2 times, whereas sequential glycosyltransferase and branching enzyme hydrolysis produced many more macropores (>50 nm), and the oil absorbency of the prepared porous starch thereby increased by 12.8 times [8]. However, the DH-15 and DH-25 with their micropores and mesopores, as well as the DH-45 with its decomposed morphology, all had higher oil absorption (Figure 1a,c), indicating that pore size is not the critical factor that determines porous properties. The higher water solubility index of DH-15 implies that has a typical pore structure, but lower transparency. Water molecules can easily enter the particles and swell, thereby enhancing the gel consistency (Figure 1d). DH-25 had better water solubility and gel consistency than the native starch, though no difference was observed in its swelling power. Furthermore, excessive starch decomposition and the rupturing of pores was observed in the DH-45 as the transparency increased. The difference was that the DH-45 showed lower swelling power and gel consistency along with an unaltered water solubility index (Figure 1d). From these results, we conclude that DH-15, which has visible micropores, exhibits typical porous starch properties, but DH-25 and DH-45 do not.

Granule integrity and starch chain interactions affect the swelling power and solubility of starch in both its amorphous and crystalline states. The increased swelling power and the solubility of porous corn starch after enzymolysis are attributed to the abundant pores extending from the surface to the center, and to the disruption of the amorphous region, which allows each granule swell more freely [33]. The amylose–lipid complex helps to decrease swelling power [34]. As expected, the DH-15 consistently exhibited these properties. However, the DH-25 and DH-45 samples exhibited decreased water solubility, swelling power, and gel consistency. The DH-45 showed the highest transparency and the lowest gel consistency, while no difference was observed in its water solubility index compared with the native corn starch. Gel consistency is commonly regarded as a good indicator of starch-based food texture, especially for food containing starch with high amylose content [35], and the swelling power of native starch is positively related to amylopectin [36]. These results suggest that AmyM hydrolysis changes the structure of starch granules and can therefore be used to prepare porous starch with different functionalities for a range of applications.

### 3.2. Crystalline Structure of the Porous Corn Starch

**Figure 2:**
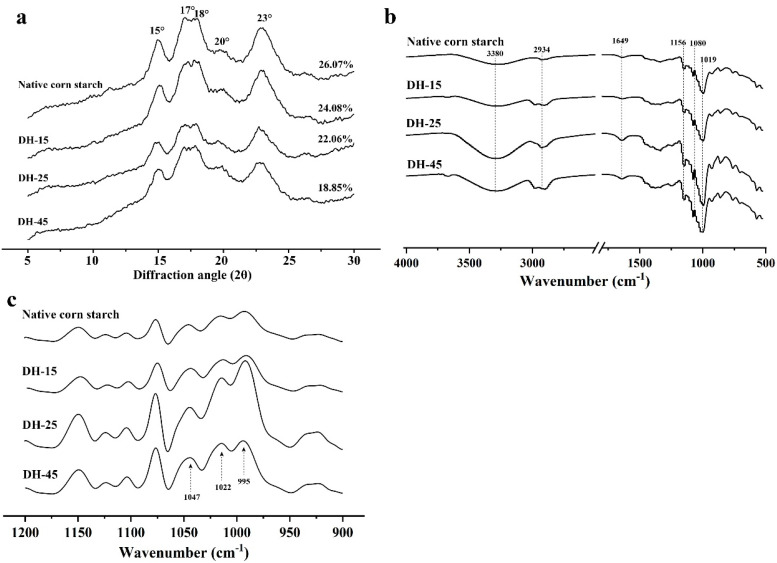
XRD spectroscopy (**a**), FTIR spectroscopy (**b**), and deconvoluted FTIR spectroscopy (**c**) of the porous corn starches. The relative crystallinity values (%) of the porous starch samples (DH−15, DH−25, and DH−45) prepared using AmyM with different degrees of hydrolysis (15, 25, and 45) are provided.

The molecular chains of amylose and amylopectin assemble in starch granules to construct multi-scale structural systems which directly affect the physicochemical properties of starch [37]. Considering the properties of porous corn starch and the possible amylopectin and amylose ratio changes resulting from AmyM treatment, we further analyzed the crystalline structures of the DH-15, DH-25, and DH-45 samples. The native corn starch and porous starch samples displayed typical A-type allomorphs with strong diffraction peaks around 15° and 23° and a doublet at 2θ of 17° and 18°. A small peak indicating the amylose–lipid crystalline complexes at 20° was also present (Figure 2a). Normally, the double helices of the crystalline lamellae can be organized in monoclinic crystalline units to form the A-type allomorph of corn starch [38], and this was observed in the current study. The AmyM treatment did not change the crystal type of the native starch, while a tendency to transform the doublet peak to single a peak was observed in the DH-25 and DH-45 samples. AmyM hydrolysis induced weaker peaks at 15°, 18°, and 23°, indicating that AmyM affects the crystalline allomorph to some degree. Compared with native corn starch granules, the crystalline area of the DH-15 decreased along with its relative crystallinity, though the amorphous area was not changed (Table 1). When the degree of hydrolysis was increased to 25 (DH-25), the relative crystallinity significantly decreased from 26.07% to 22.06%, and the amorphous area and the crystalline area both decreased as well (Figure 2a and Table 1). This could indicate that AmyM affects both crystalline and amorphous regions, and thereby decreases the proportion of the crystalline structure. However, the total peak area and the crystalline and amorphous areas increased in the DH-45 sample, and the peak at 20° was enhanced (Table 1).

The enzymatic hydrolysis of raw starch preferentially occurs in the amorphous region and leads to an increase in the relative proportion of the crystalline region [39], thereby allowing increased swelling power and solubility [33]. In contrast, the hydrolysis of the crystalline and amorphous regions of corn starch granules by AmyM produces porous corn starch with reduced relative crystallinity, indicating that AmyM facilitates interior granule decomposition in supramolecular structures, including the crystalline and amorphous regions of starch. Native starch granules typically have a crystallinity which varies from 15% to 45%, and the double helices which are formed from the external chains of amylopectin crystallize into an amorphous lamella and then a thin lamella, which together build up semi-crystalline rings in starch granules that are about 100 and 400 nm thick [40]. Generally, the crystalline regions within starch granules resist the hydrolysis of RSDEs, resulting in a low degree of hydrolysis (Table S1). AmyM induces the dual hydrolysis of both the crystalline and amorphous regions, which not only explains the 95% degree of hydrolysis observed in the corn starch granules, but also raises the possibility that AmyM hydrolysis promotes the formation of sub-microcrystalline structures within ordered-starch molecules, which in turn contribute to the formation of porous starch with the same level of water solubility as native starch, but decreased gel consistency. The location and the state of the amylose within the granule indicate that amylose mainly enriches the granule near the surface, and some amylose chains cross-link with amylopectin chains [40]. By combining the hydrolytic properties of AmyM and the structural features of porous starch granules, we deduced that the preferential hydrolysis of AmyM toward amylose disrupts the surface structure of starch at the initial stage, thereby promoting the formation of small pores and then affecting the cross-linking of amylose and amylopectin, which contributes to a reduction in crystallinity. In addition to inducing the transition of DH-25, the preferential hydrolysis of amylopectin destroys amylopectin clusters with new external chains, and thus random amylose may form helix moieties in DH-45. Although these structures are not involved in extended crystalline arrays, they may somehow pack into imperfect crystalline structures [40]. Oligosaccharides such as maltohexaose are able to co-crystallize in the presence of longer chains [41]. AmyM is a maltohexaose-forming amylase which hydrolyzes gelatinized starch into maltooligosaccharides with maltohexaose as the major component [15], and this may also have contributed to the interactions of the chains. These results indicate that the modification of starch chains using AmyM hydrolysis promotes molecular rearrangement within starch granules and the formation of ordered structures.

### 3.3. Short-Range Ordered Structure of Porous Corn Starch Detected Using FTIR

The FTIR spectra of the native corn starch, DH-15, DH-25, and DH-45 were analyzed. A is shown in Figure 2b, the O-H stretching vibration and the C-H deformation of the glucose unit typical in starch, with characteristic peaks at 3380 cm^−1^ and 2934 cm^−1^, were significantly enhanced in the DH-15, DH-25, and DH-45 compared with the control, and the value of the peak at 1649 cm^−1^, which we attribute to the bending vibration of the O-H in the water that was absorbed in the amorphous regions of starch [42], was also increased in the DH-25 and DH-45. The peak intensity, including the C-O stretching vibrations at 1019 cm^−1^ and the C-H and C-O-H bending at 1080 cm^−1^ and 1019 cm^−1^ [43], respectively, were enhanced as DH the increased. The increased peak intensity may be due to the chain interactions resulting from the additional exposed hydroxyl groups in the starch samples after AmyM hydrolysis. This would be consistent with the properties of the porous corn starch prepared using α-amylase and amyloglucosidase [44]. The peak intensity of the DH-45 at 3400 cm^−1^ was obviously decreased compared with that of the DH-25, indicating that the exposed hydroxyl groups of the DH-45 promote the formation of ordered molecular structures via hydrogen bond interactions.

To understand the short-range molecular orders of the DH-15, DH-25, and DH-45, the deconvoluted FTIR spectra at the 1047 and 1022 cm^−1^ regions associated with the crystalline and amorphous phases of the starch were analyzed (Figure 2c). A decreased absorbance ratio of 1047/1022 cm^−1^ was observed in the DH-15 (this was lower than in the native corn starch), indicating that the disordered structure of the porous starch was produced during the initial stage (consistent with our previous assumptions). A similar result was also observed in the porous starch prepared using glucoamylase [5]. Whereas glucoamylase mainly targets the amorphous structures, AmyM disrupts the crystalline region as well during the initial stage. A decreased absorbance ratio of 1047/1022 cm^−1^ and a lower absorbance ratio of 1022/995 cm^−1^ were observed in the DH-25, and these represent the moderate molecular order of the double helices of the starch granules. Interestingly, the DH-45 displayed increased absorbance ratios of 1047/1022 cm^−1^ and 1022/995 cm^−1^ compared with the DH-25 (Table 2). The band at 1022 cm^−1^ is responsible for the less ordered structures, and the band at 995 cm^−1^ with the C-OH bending vibrations indicates the strength of the interchain interactions caused by hydrogen bonding in the case of amylose [45]. We deduced that imperfect crystalline phases with more short-range ordered structures are formed in DH-45 by the enhancement of starch chain interactions, and we preliminarily define these as sub-microcrystalline structures. The rearrangement of starch molecular chains resulting in the formation of short-range ordered structures can be achieved using extrusion technology or ultrasonic and enzymatic treatments [44,46]. From the results concerning the crystalline and short-range ordered structures of the porous corn starch, we concluded that the hydrolysis of starch granules using AmyM (achieved via the selective hydrolysis of amylose and amylopectin at different stages) promotes the formation of short-range ordered structures via chain–chain or chain–water interactions, which benefit from the additional exposed inner surfaces and nanoscale holes in the lamellar structures.

### 3.4. Thermal Properties

**Figure 3:**
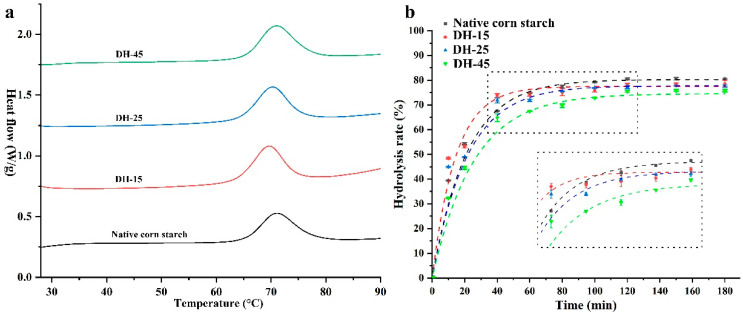
Thermal properties (**a**) and digestive evaluation (**b**) of the porous corn starch samples. The DH-15, DH-25, and DH-45 were prepared using AmyM and have 15, 25, and 45% degrees of hydrolysis, respectively.

Considering the water migration properties of the porous corn starch obtained from the pasting analysis, the gelatinization properties of the porous corn starch were further analyzed by using DSC thermograms to detect possible changes in the physical states of the starch slurry. As is shown in Figure 3a and Table 3, the native corn starch displayed similar gelatinization enthalpy (Δ*H_g_*) to the DH-15 and DH-25, whereas the Δ*H_g_* of the DH-45 was significantly higher than that of the native starch. The DH-25 was regarded as the product of an intermediate stage of AmyM hydrolysis, and this was consistent with the increased Δ*H_g_* values and the increased temperature range. The effect of annealing may have been to enhance the degree of molecular order in the chain rearrangements [47]; this would be consistent with the XRD results (Figure 2a). The DH-15 and DH-25 exhibited lower temperature ranges (*T_c_*-*T_o_*) than the control, and this may have been a result of the rearranging of part of the double helix due to the presence of excess water [48]. Maltogenic α-amylase hydrolysis has been shown to decrease gelatinization temperatures and Δ*H_g_* due to the hydrolysis of double helices, and transglucosidase in combination with maltogenic α-amylase and β-amylase can lower transition temperatures due to the reduction of longer chains, which leads to a greater number of shorter double helices and thereby decreases thermal resistance [13,49]. The gelatinization enthalpy reflects the thermal energy of the destruction of crystallites and the dissociation and unraveling of double helices in crystalline and non-crystalline segments [49]. The thermal properties of the porous starch samples indicate that the AmyM treatment led to the formation of short-range ordered structures via the preferential hydrolysis of starch chains. However, more evidence is needed to confirm this.

### 3.5. Digestion Evaluation of the Porous Corn Starch

Porous starch can be used in a wide variety of food-related applications, raising questions concerning how the porous function of porous starch might affect digestibility. Hence, we analyzed the digestibility curves of the enzymatically prepared porous starches, and the parameters include the equilibrium concentration of the hydrolyzed starch (*C*_∞_), the kinetic constant (*k*), the area under the hydrolysis curve after 180 min (AUC 180), the hydrolysis index (HI), and the estimated glycemic index (eGI). As is shown in Figure 3b, no differences were observed during the early hydrolysis stage, and the porous starch samples showed lower hydrolysis rates at the very end of the digestion reaction. The porous starch prepared using amylase or amyloglucosidase displayed accelerated digestion rates, which contributed to the specific pore size-mediated access and the exposure of the amorphous areas to the exogenous enzymes [39]. In this study, none of the AmyM-treated porous corn starches with diverse pore sizes had any significant effect on enzymatic digestion during the initial stage, though restrained digestion with limited glucose release was observed during the later stage, as was verified by the digestibility constant (*k*) end point values (*C*_∞_) (Table 4). An 18.9% decrease in the *C*_∞_ value was observed in the DH-45, indicating the potential restrictive digestibility of the starch granules [39]. Decreased AUC 180, HI, and eGI values were also observed in the DH-15, DH-25, and DH-45, indicating lower glucose release [26]. The minor decreases in the hydrolysis rates of the porous starches produced via AmyM hydrolysis also agreed with the increase in RS observed in the DH-25 and DH-45 (Table 4). Our results show that the porous corn starch samples prepared via AmyM hydrolysis had no positive effects on digestibility at lower hydrolysis rates. 

Hydrolytic enzymes such as amyloglucosidase AMG, fungal α-amylase AM, and cyclodextrin glycosyltransferase CGTase have been shown to affect the amorphous and crystalline domains of starch, and the presence of pores on the surface increases the exposure of the granule to subsequent enzymatic digestion [50]. However, opposite results have also been observed following treatment with AM, CGTase, and branching enzyme BE, which offer significant resistance to enzymatic hydrolysis via the accumulation of more organized residues from the hydrolysis of the amorphous region and thereby impede enzyme accessibility [51]. These results indicate that porous starch obtained from enzyme treatment harbors different digestive properties, and this is important for its application in the food industry considering the desirability of low glycemic food. From this study, we conclude that AmyM disrupts the amorphous and crystalline regions of corn starch granules and produces visible micropores in the crystalline region as well as amylose chains, and these promote molecular rearrangement by regulating the ratio of amylose and amylopectin and lower the digestive rate.

From the above results, we suggest that modification via AmyM can induce structural changes in raw starch granules. AmyM hydrolyzes the amylose located at the surface and in the crystalline and amorphous regions during the initial stage (DH-15). The micropores that are produced allow AmyM to display ‘inside-out’ behavior toward corn starch granules. AmyM preferentially hydrolyzes the amylopectin in the crystalline region during the later stage (DH-45) via intermediate state DH-25 and exposes the granules to subsequent enzymatic binding. The associations of dissociative amylose chains and the interactions of maltohexaose-like oligosaccharides with long chains promote the formation of sub-microcrystalline structures via chain rearrangement, and the resulting short-range ordered structures retain the characteristics of native starch granules.

## 4. Conclusions

In the present study, solid granular corn starch was treated using a maltohexaose-forming α-amylase (AmyM) below the gelatinization temperature, and this produced porous starch samples with porous surface and internal morphology via the decomposition of the amorphous and crystalline regions. Lower relative crystallinity, unstable granules, and the formation of short-term ordered structures by the potential amylose chain interactions in the amorphous region were observed. AmyM-mediated structural regulation conferred the porous properties of the samples, decreased the *C*_∞_ values, increased the RS content, and lowered the AUC, HI, and eGI values. This has important implications for the application of AmyM in the preparation of porous starch. From this study, we conclude that the amylose-mediated ordered structure created via uniform enzymolysis is also the rate-limiting factor for digestion (excepting surface properties, pore size, and the amorphous region). However, more details concerning the structural features of porous starch are needed. Overall, modification via maltooligosaccharide-producing amylase at a low temperature offers an attractive alternative for obtaining porous starch granules that can be used in a variety of foods applications.

## Figures and Tables

**Table 1:** Structural parameters of porous corn starch from XRD analysis.

Sample	Native Corn Starch	DH-15	DH-25	DH-45
Total peak area	398,946 ± 2177 a	387,184 ± 4574 b	299,730 ± 2889 c	385,044 ± 6982 b
crystalline area	103,986 ± 1092 a	93,239 ± 291 b	66,106 ± 239 d	72,578 ± 539 c
Amorphous area	294,960 ± 686 b	293,945 ± 3444 b	233,625 ± 2598 c	312,466 ± 5161 a
Relative crystallinity (%)	26.07 ± 0.19 a	24.08 ± 0.19 b	22.06 ± 0.31 c	18.85 ± 0.17 d

Values followed by different letters within a single row differ significantly (*p* < 0.05).

**Table 2:** Structural parameters of porous corn starch prepared using AmyM.

Sample	Native Corn Starch	DH-15	DH-25	DH-45
Relative amylose content (%)	38.52 ± 0.17 b	34.96 ± 0.08 d	36.44 ± 0.18 c	39.2 ± 0.33 a
*d* (nm)	9.40 ± 0.02 a	9.18 ± 0.03 b	9.07 ± 0.02 c	8.55 ± 0.01 d
*d*_a_ (nm)	3.73 ± 0 b	3.67 ± 0.02 c	3.64 ± 0.03 c	3.83 ± 0.01 a
*d*_c_ (nm)	5.67 ± 0.01 a	5.51 ± 0.01 b	5.43 ± 0.04 c	4.72 ± 0.02 d
*A*_peak_ (a.u.)	0.27 ± 0.00 a	0.28 ± 0.02 a	0.24 ± 0.01 b	0.22 ± 0.01 c
1047 cm^−1^/1022 cm^−1^	0.725 ± 0.001 a	0.715 ± 0.003 b	0.604 ± 0.001 c	0.683 ± 0.007 d
1022 cm^−1^/995 cm^−1^	0.866 ± 0.008 b	0.872 ± 0.004 b	0.820 ± 0.005 c	0.943 ± 0.008 a

*d*: average thickness of semi-crystalline lamellae; *d*_c_: average thickness of crystalline lamellae; *d*_a_: average thickness of amorphous lamellae; *A*_peak_: area of the scattering peak. Values followed by different letters within a single row differ significantly (*p* < 0.05).

**Table 3:** Pasting and thermal properties of gelatinized porous corn starch.

Sample	Native Corn Starch	DH-15	DH-25	DH-45
*T*_c_-*T*_o_ (°C)	11.0 ± 0.2 a	7.0 ± 0.2 b	6.7 ± 0.3 b	9.6 ± 0.2 c
Δ*H_g_* (J/g)	11.0 ± 0.3 b	10.5 ± 0.2 b	11.0 ± 0.2 b	12.1 ± 0.1 a

*T*_c_*-T*_o_: temperature range; Δ*H_g_*: gelatinization enthalpy. Values are means ± SD. Values followed by different letters within a single row differ significantly (*p* < 0.05).

**Table 4:** Effects of AmyM treatment on starch digestibility and expected glycemic index.

Sample	*C*_∞_	*k*	AUC 180	HI	eGI	RS (%)
Native starch	25.16 ± 0.76 a	0.013 ± 0.001 c	2732 ± 2 a	100.00 ± 0.09 a	94.40 ± 0.08 a	14.04 ± 0.05 b
DH-15	21.44 ± 0.47 b	0.018 ± 0.001 ab	2673 ± 24 b	97.84 ± 0.90 b	92.54 ± 0.77 b	14.41 ± 0.24 b
DH-25	21.17 ± 0.48 bc	0.017 ± 0.001 b	2615 ± 7 c	95.72 ± 0.27 c	90.71 ± 0.23 c	15.93 ± 0.07 a
DH-45	20.39 ± 0.30 c	0.019 ± 0.001 a	2620 ± 3 c	95.90 ± 0.12 c	90.86 ± 0.10 c	16.02 ± 0.27 a

*C*_∞_: equilibrium concentration; *k*: kinetic constant; HI: hydrolysis index; AUC 180: area under curve after 180 min; eGI: estimated glycemic index. Values are the means ± SD. Values followed by different letters within a single column differ significantly (*p* < 0.05).

## Data Availability

The data used to support the findings of this study can be made available by the corresponding author upon request.

## Supplementary Materials

The following supporting information can be downloaded at: https://www.mdpi.com/article/10.3390/foods12173157/s1, Table S1: Specific activities and hydrolysis rates of AmyM and some representative amylases toward soluble starches and raw starches [3,52,53,54,55,56].
