# Supplementary material for: Structural and Functional Properties of Porous Corn Starch Obtained by Treating Raw Starch with AmyM
Source: Foods. 2023 Aug 22;12(17):3157. doi: 10.3390/foods12173157 (PMC10486553; doi:10.3390/foods12173157)
Supplement: Supplementary file 1 [file foods-12-03157-s001.zip › foods-2548255-supplementary.pdf]

Table S1. Specific activities and hydrolysis rates of AmyM and some representative amylases toward soluble starches and raw starches.

Sources	Specific activity (U/mg)		Degree of hydrolysis (%)	Enzyme amount (U/mg starch)	Hydrolysis condition	Reference
	Soluble starch	Raw corn starch	Raw corn starch			
B. subtilis BF7658		78.15	65.5%, 6h	0.1	40°C, pH 6.0	[52]
Pontibacillus sp. ZY (AmyZ1)	14815	9056	47% , 4 h	1.0	30°C, pH 7.0	[53]
Penicillium oxalicum (PoGA15A)	81.2	23.7	65.5%, 72 h [a]	0.05	40°C, pH 4.5	[54]
Geobacillus sp. 4j (Gs4j-amyA)	8600	n.d.	36%, 4 h	0.5	65°C, pH 5.5	[55]
Bacillus licheniformis (BLA)	276	15.7	58% , 5 h	11.5	60°C, pH 6.5	[55]
Bacillus (NOVO-Termamyl 300L)	1050	0.54	n.d.	n.d.	n.d.	Novozymes
Rhizomucor sp. (RA)	n.d.	n.d.	75%, 96 h [b]	15.5	32°C, pH 4.5	[56]
Corallococcus sp. EGB (AmyM)	14000	1110	63%, 4 h	5.0	45°C, pH 6.0	[3]

The used concentration for determination of the degree of hydrolysis of the raw corn starch was 30%. [a] indicates 15%, [b] indicates 31%. n.d. mean not determined.

Reference:

3. Zhang, L.; Zhong, L.; Wang, J.; Zhao, Y.; Zhang, Y.; Zheng, Y.; Dong, W.; Ye, X.; Huang, Y.; Li, Z. Efficient hydrolysis of raw starch by a maltohexaose-forming α-amylase from *Corallococcus* sp. EGB. *LWT* **2021**, 152, 112361.

52. Tang, S.; Xu, T.; Peng, J.; Zhou, K.; Zhu, Y.; Zhou, W.; Cheng, H.; Zhou, H. Overexpression of an endogenous raw starch digesting mesophilic α-amylase gene in *Bacillus amyloliquefaciens* z3 by in vitro methylation protocol. *J. Sci. Food Agr.* **2020**, *100*, 3013-3023.

53. Fang, W.; Xue, S.; Deng, P.; Zhang, X.; Wang, X.; Xiao, Y.; Fang, Z. Amyz1: A novel α-amylase from marine bacterium *Pontibacillus* sp. Zy with high activity toward raw starches. *Biotechnol. Biofuels* **2019**, *12*, 1-15.

54. Xu, Q.-S.; Yan, Y.-S.; Feng, J.-X. Efficient hydrolysis of raw starch and ethanol fermentation: A novel raw starch-digesting glucoamylase from penicillium oxalicum. *Biotechnol. Biofuels* **2016**, *9*, 1-18.

55. Jiang, T.; Cai, M.; Huang, M.; He, H.; Lu, J.; Zhou, X.; Zhang, Y. Characterization of a thermostable raw-starch hydrolyzing α-amylase from deep-sea thermophile *Geobacillus* sp. *Protein Expres. Purif.* **2015**, *114*, 15-22.

56. Tawil, G.; Viksø-Nielsen, A.; Rolland-Sabaté, A.; Colonna, P.; Buléon, A. In depth study of a new highly efficient raw starch hydrolyzing α-amylase from *Rhizomucor* sp. *Biomacromolecules* **2011**, *12*, 34-42.
